# The Effects of Different Oxytetracycline and Copper Treatments on the Performance of Anaerobic Digesters and the Dynamics of Bacterial Communities

**DOI:** 10.1155/2018/1897280

**Published:** 2018-07-05

**Authors:** Yun Zhang, Xin Ke, Wei Sun, Guangcai Zhang, Xiaodan Gao, Haijun Zhang, Weiyun Wang

**Affiliations:** ^1^Northeast Key Laboratory of Arable Land Conservation and Improvement, Ministry of Agriculture, National Engineering Laboratory for Efficient Utilization of Soil and Fertilizer Resources, College of Land and Environment, Shenyang Agricultural University, Shenyang 110866, China; ^2^College of Energy and Environment, Shenyang Aerospace University, Shenyang 110136, China

## Abstract

Oxytetracycline and copper are the common residues in animal manures. Meanwhile, anaerobic digestion is considered as a clean biotechnology for the disposal of animal manures. In this paper, the performance of anaerobic digesters and the dynamics of bacterial communities under the different treatments of oxytetracycline and copper were discussed. The parameters of methane production and pH values were studied to reflect the performance of anaerobic digester. Results showed that the changes of methane production and pH values were not obvious compared with the control. This means that the treatments of oxytetracycline and copper almost have no effects on the performance of anaerobic digesters. This phenomenon might be due to the chelation reaction between oxytetracycline and copper. This chelation reaction might reduce the toxicity of oxytetracycline. The study on the dynamics of bacterial communities was based on the polymerase chain reaction-denaturing gradient gel electrophoresis (PCR-DGGE) method. Results indicated that the bacterial communities had significant differences under the different treatments of oxytetracycline and copper. Uncultured* Bacteroidetes* bacterium (CU922272.1) and uncultured* Bacteroidetes* bacterium (AB780945.1) showed adaptability to the different treatments of oxytetracycline and copper and were the dominant bacterial communities.

## 1. Introduction

With the development of livestock, the residual antibiotics and heavy metals in animal manure have attracted more and more world-wide attention. This residual phenomenon is due to the low bioavailability of antibiotics and heavy metals which are widely added to the feeds in order to control diseases and enhance the growth of livestock animals [1–4]. Heuer et al. [5] found that 30–90% of the antibiotics as feeding additives were excreted through urine and manures. The residual metals in manure were also considerable [6, 7]. Oxytetracycline and copper are the common residues in animal manures. It was found that the residual oxytetracycline in cow manure ranged from 0.32 mg/kg to 59.59 mg/kg [8]. The residual copper could reach up to 481.5 mg/kg in cattle manures [9]. These residual antibiotics and heavy metals can cause the potential threats to environment. Therefore, it is necessary to find appropriate methods to solve the problems of residual oxytetracycline and copper in animal manure.

Animal manures belong to the organic wastes. The common methods of the disposal of manure include aerobic composting and anaerobic digestion [10–12]. Compared to other methods, anaerobic digestion belongs to a clean biotechnology which can produce biogas [13]. Meanwhile, the use of biogas can reduce the consumption of fossil fuels and the emissions of greenhouse gases [14, 15]. The residual digestate after anaerobic digestion can be used as an improved fertilizer [16].

The performance of anaerobic digester can be reflected by methane production and pH values [17, 18]. Furthermore, anaerobic digestion is considered to be a biological process which involves many classes of bacteria. It consists of four stages: hydrolysis, acidogenesis, acetogenesis, and methanogenesis [19, 20]. Bacterial communities show dynamics during the process of anaerobic digestion. Bouallagui et al. [21] studied the dynamics of bacterial communities in a two-phase anaerobic bioreactor. They found that the species composition of bacterial communities had very significant changes during the process of anaerobic digestion. Patil et al. [22] studied the dynamics of microbial community via polymerase chain reaction-denaturing gradient gel electrophoresis (PCR-DGGE) and found that* Firmicutes* and uncultured bacteria were the dominant genera in the mesophilic digesters treating with piggery wastewater. However, there is still short of the study on the dynamics of bacterial communities under the treatments of oxytetracycline and copper during anaerobic digestion.

The objectives of this study were to evaluate the performance of anaerobic digesters and discuss the dynamics of bacterial communities under the treatments of oxytetracycline and copper during the anaerobic digestion of cow manure. Methane production and pH values were measured and analyzed to reflect the performance of anaerobic digestion. Furthermore, the PCR-DGGE method was used to discuss the dynamics of bacterial communities.

## 2. Materials and Methods

### 2.1. Experimental Set-Up and Analytical Methods

Cow manure samples were taken from the surrounding countryside Shenyang City, China. Then the samples were stored in a refrigerator at 4°C before used. The physical and chemical properties of samples were as follows: total solids, 25.67%; pH value, 8.26; volatile organic acids, 878.4 mg l^−1^; and organic carbon, 42.75%. Laboratory-scale anaerobic digesters (1 L) were prepared. Then each digester was added with 200 g cow manure. Different amounts of oxytetracycline and copper (dose as CuSO_4_) were added to each digester (Table 1). In this experiment, the fermentation broth of digested cow manure was used as the inoculum. Each anaerobic digester was inoculated with 200 ml inoculum, then continuously stirred, and maintained at a mesophilic condition of 37°C in a water bath. All the experiments were performed in triplicate. Methane production was measured by gas chromatography (GC-14B, Shimadzu, Japan). The pH values were determined using a hand-held pH meter.

### 2.2. DNA Extraction and PCR-DGGE

Total genomic DNA of manure samples was extracted on days 1, 15, and 50, respectively. In this experiment, the primers 341F with 40 bp GC-clamps and 907R were used for the polymerase chain reaction amplification [23]. The operations of DNA extraction and PCR-DGGE were carried out as the previous study [24], but with some modifications that the electrophoresis was performed in a 7 L 1×TAE buffer at 60°C for 6 h at 180 V in this experiment.

### 2.3. Sequencing and Phylogenetic Analysis

The selected DGGE bands were reamplified and electrophoresed to confirm the mobility and then transported to Beijing Huada Gene Company (Beijing, China) for sequencing. Through the CLUSTAL X and MEGA 4.0, the phylogenetic tree was built via the neighbor-joining method [25].

### 2.4. Nucleotide Sequence Accession Numbers

Nucleotide sequences were deposited in the NCBI nucleotide sequence databases to get the accession numbers: KM491540-KM491545.

## 3. Results and Discussion

### 3.1. The Performance of Anaerobic Digesters

#### 3.1.1. Methane Production

**Figure 1:**
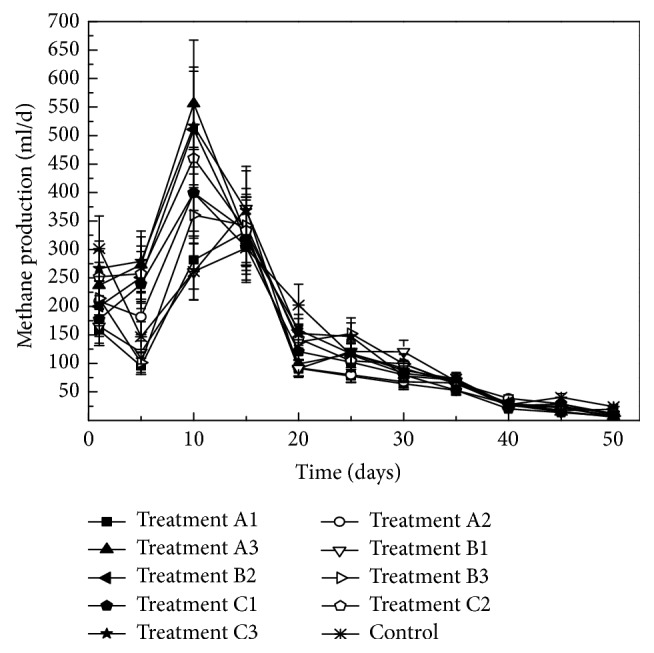
Variations of methane production with time under different oxytetracycline and copper treatments.

Methane production under the different treatments of oxytetracycline and copper is present in Figure 1. The highest methane production under treatments A1, A2, A3, B1, B3, B4, C1, C2, C3, and the control was 331.9 ml (day 15), 399.5 ml (day 10), 556.2 ml (day 10), 371.7 ml (day 15), 510.5 ml (day 10), 360.5 ml (day 10), 399.5 ml (day 10), 460.3 ml (day 10), 516.8 ml (day 10), and 302.7 ml (day 15), respectively. At the first five days, the process of anaerobic digestion was not stable. Then the methane production increased until the 10th day. Compared with the control, the curve of methane production had nonsignificant differences after the 25th day. This might be caused by the chelation reaction between oxytetracycline and copper. Pouliquen and Le Bris [26] found that oxytetracycline was likely to form complexes with mineral cations. Moreover, Hassan et al. [27] reported that oxytetracycline could form the copper-oxytetracycline chelates. Previous studies had found that oxytetracycline had inhibition on methane production during the anaerobic digestion [28, 29]. However, the chelation reaction between oxytetracycline and copper might reduce the toxicity of oxytetracycline. Therefore, the treatments of oxytetracycline and copper had little effect on the methane production.

#### 3.1.2. The pH Values

**Figure 2:**
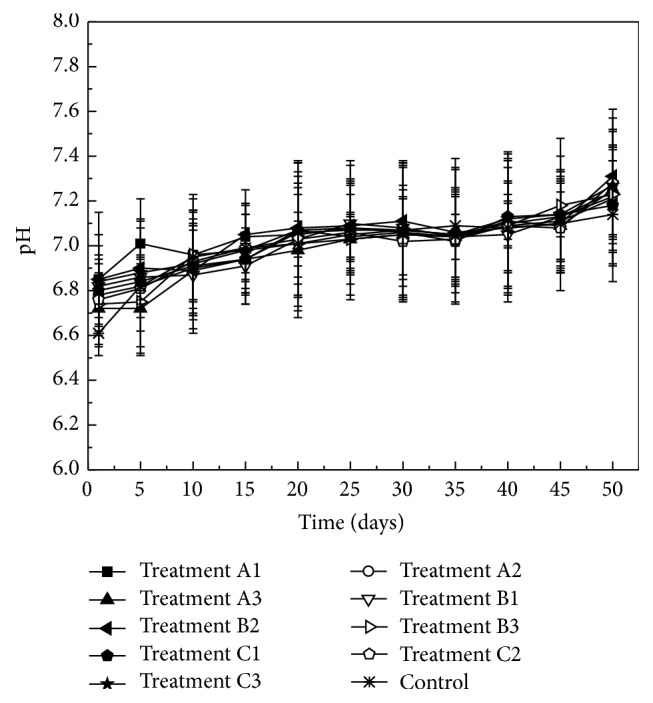
Variations of pH value with time under different oxytetracycline and copper treatments.

The changes of pH values during the anaerobic digestion are shown in Figure 2. The pH values under all treatments ranged from 6.61 to 7.31. This range belongs to the optimal pH values to produce maximal biogas yield. Throughout the process of anaerobic digestion, pH values gradually increased. This might be due to the continuous stirring which could make cow manure continued dissolution. The highest pH values which were all present at the end of the anaerobic digestion were 7.21 (treatment A1), 7.24 (treatment A2), 7.25 (treatment A3), 7.22 (treatment B1), 7.31 (treatment B2), 7.23 (treatment B3), 7.18 (treatment C1), 7.28 (treatment C2), 7.27 (treatment C3), and 7.14 (control). As is shown in Figure 2, the pH values under all treatments did not present significant differences compared with the control. This means that the treatments of oxytetracycline and copper almost have no significant effects on the pH values during the process of anaerobic digestion of cow manure.

### 3.2. Dynamics of Bacterial Communities

**Figure 3:**
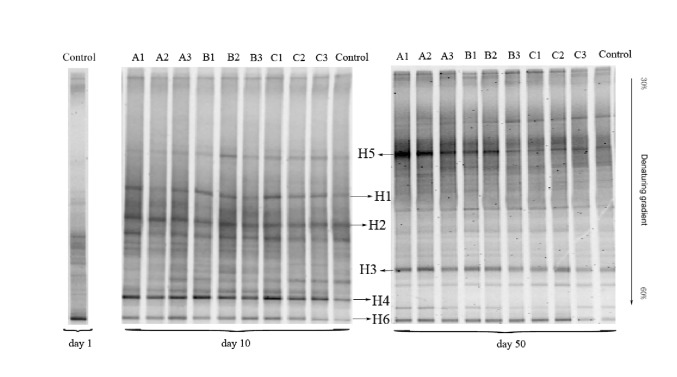
Denaturing gradient gel electrophoresis (DGGE) fingerprints of bacterial 16S-rRNA gene fragments of cow manure samples under different oxytetracycline and copper treatments at day 1, day 10, and day 50.

Dynamics of bacterial communities under the different treatments of oxytetracycline and copper are present via the DGGE fingerprints in Figure 3. The DGGE band patterns showed significant differences and clear changes under different treatments. Bands H1, H2, and H4 were detected at day 10. However, they disappeared at day 50. Band H3 could only be observed at day 50. Bands H5 and H6 were present under all the treatments of oxytetracycline and copper during the whole process of anaerobic digestion. They were the dominant bacterial communities.

**Figure 4:**
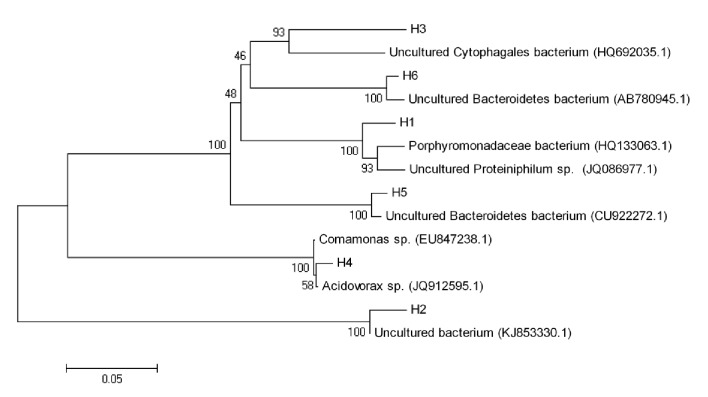
Phylogenetic tree of the bacterial 16S-rRNA gene sequences compared with known sequences from Genbank.

Although band H5 was not shown at the control DGGE bands of day 1, it appeared at the control DGGE bands of day 10 and day 50. In contrast, band H6 was shown at all DGGE bands. This indicated that band H6 seemed to play as the functional bacteria. Through the sequence similarity analysis by the BLAST program, these six bacterial sequences were conducted by homology comparison (Table 2). The phylogenetic tree was established in Figure 4. Results showed that Band H2 had 98% similarity to uncultured bacterium (KJ853330.1). Band 4 was closely related to* Acidovorax* sp. (JQ912595.1). Hoshino et al. [30] found that* Acidovorax* sp. played an important role in denitrification. Band 3 had high similarity to uncultured* Cytophagales* bacterium (HQ692035.1). Band H1 shared 95% similarity with* Porphyromonadaceae* bacterium (HQ133063.1). These three kinds of bacteria all could be detected in the anaerobic digestion [31, 32]. Band H5 had 99% similarity to uncultured* Bacteroidetes* bacterium (CU922272.1). Band H6 was closely related to uncultured* Bacteroidetes* bacterium (AB780945.1). Riviere et al. [33] reported that uncultured* Bacteroidetes* bacterium (CU922272.1) existed in the mesophilic anaerobic digestion of municipal sewage sludge. Uncultured* Bacteroidetes* bacterium (AB780945.1) could be found in a full-scale mesophilic anaerobic completely stirred tank reactor during the anaerobic digestion of untreated corn straw [34].

In this experiment, it could be concluded that uncultured* Bacteroidetes* bacterium (CU922272.1) and uncultured* Bacteroidetes* bacterium (AB780945.1) showed adaptability to the different treatments of oxytetracycline and copper and were the dominant bacterial communities during the anaerobic digestion under the treatments of oxytetracycline and copper.

## 4. Conclusions

This study discussed the performance of anaerobic digesters and the dynamics of bacterial communities under different treatments of oxytetracycline and copper. Results indicated that methane production and pH values were hardly affected compared with the control. This might be due to the chelation reaction between oxytetracycline and copper. The reaction might reduce the toxicity of oxytetracycline. Meanwhile, uncultured* Bacteroidetes* bacterium (CU922272.1) and uncultured* Bacteroidetes* bacterium (AB780945.1) were the dominant bacterial communities during the anaerobic digestion under the treatments of oxytetracycline and copper. This research can help to optimize the performance of anaerobic digestion and the structure of bacterial community for increasing the biogas production and reducing the pollution of residues.

## Figures and Tables

**Table 1:** Different treatments of oxytetracycline and copper in the laboratory-scale anaerobic digesters.

Treatments	Oxytetracycline dry weight (mg/kg)	Copper (dose as CuSO_4_) dry weight (mg/kg)
A1	20	100
A2	20	200
A3	20	300
B1	50	100
B2	50	200
B3	50	300
C1	100	100
C2	100	200
C3	100	300
Control	0	0

**Table 2:** Closest relatives of the bacterial 16S-rRNA gene sequences.

DGGE band	Closest GenBank Relative (accession number)	Sequence homology (%)	Accession Number
H1	*Porphyromonadaceae* bacterium (HQ133063.1)	95%	KM491540
H2	Uncultured bacterium (KJ853330.1)	98%	KM491541
H3	Uncultured *Cytophagales* bacterium (HQ692035.1)	90%	KM491542
H4	*Acidovorax* sp. (JQ912595.1)	99%	KM491543
H5	Uncultured *Bacteroidetes* bacterium (CU922272.1)	99%	KM491544
H6	Uncultured *Bacteroidetes* bacterium (AB780945.1)	98%	KM491545
